# Editor’s Note

**Published:** 2011-02

## 2010 Reviewers of the Year

Like all peer-reviewed journals, *EHP* relies on the diligence and integrity of experts to help determine the quality and impact of papers submitted for possible publication. In 2010 *EHP* received over 1,450 papers, and about 550 of those papers were sent by our Associate Editors to at least two anonymous peer reviewers for evaluation. *EHP* published 268 papers in 12 issues during 2010, and the journal is very grateful for the time and effort of the more than 1,000 reviewers who assisted us last year. A list of those reviewers is available on the journal’s website (http://ehponline.org/article/info:doi/10.1289/ehp.119-a59). In this issue, *EHP* recognizes its top 12 Reviewers of the Year. These are individuals who reviewed at least five papers during the year and received excellent ratings for the timeliness and quality of their reviews by the Associate Editor who handled the peer-review process. They are Carol Angle, Adrian Barnett, Joe Braun, Jane Clougherty, Adrienne Ettinger, Matthew Longnecker, John Meeker, Sumi Mehta, David Savitz, Leonardo Trasande, Roberta White, and Judith Zelikoff. We congratulate these reviewers and thank the hundreds of others who contributed to the success of *EHP* in 2010.

## Figures and Tables

**Figure:**
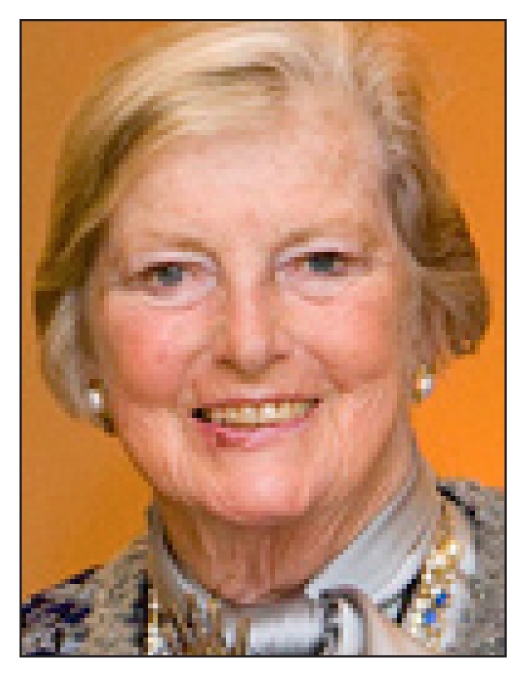
Carol Angle

**Figure:**
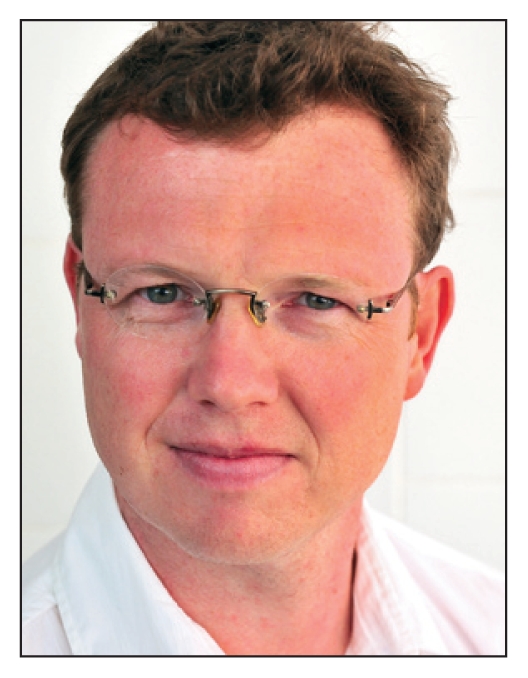
Adrian Barnett

**Figure:**
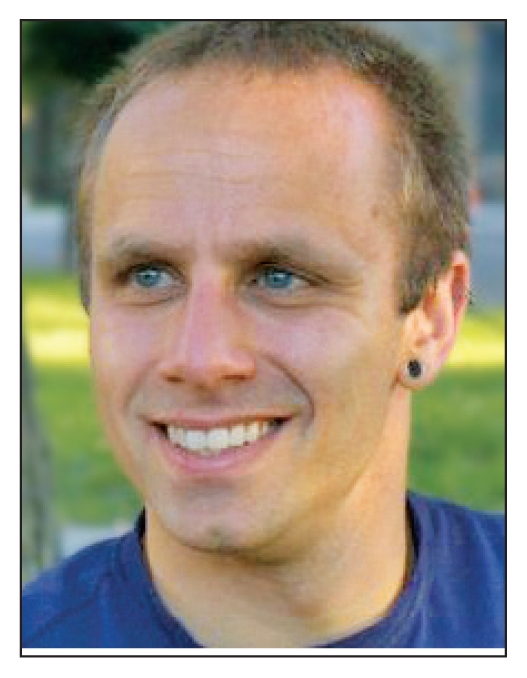
Joe Braun

**Figure:**
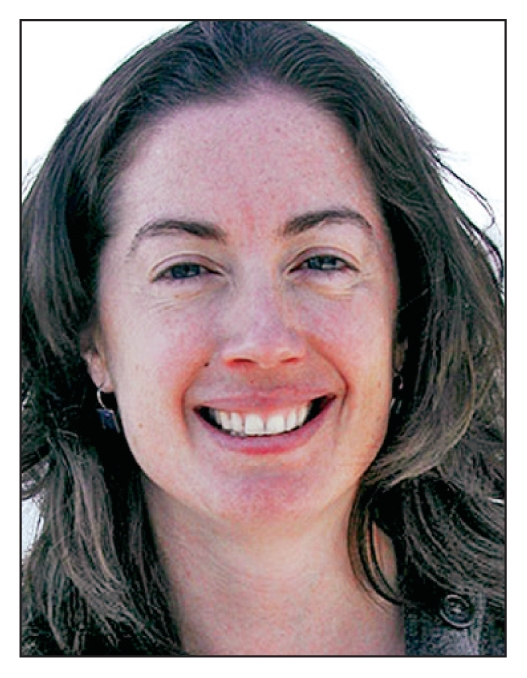
Jane Clougherty

**Figure:**
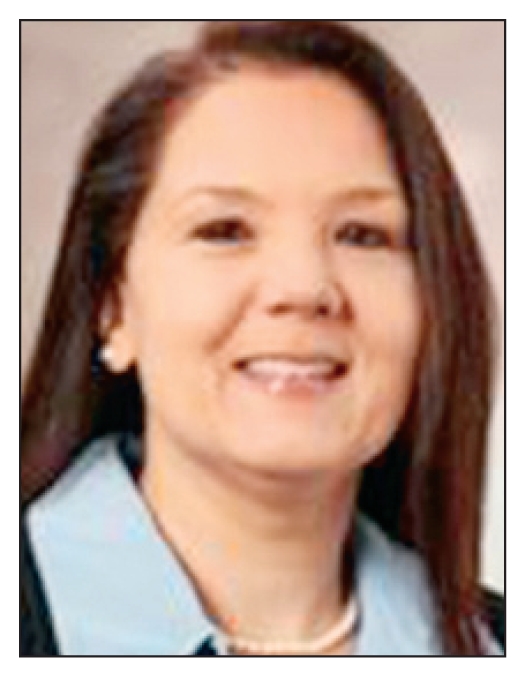
Adrienne Ettinger

**Figure:**
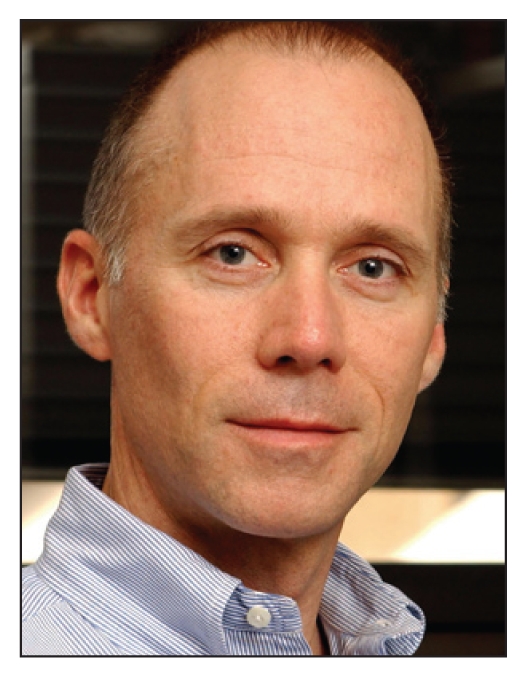
Matthew Longnecker

**Figure:**
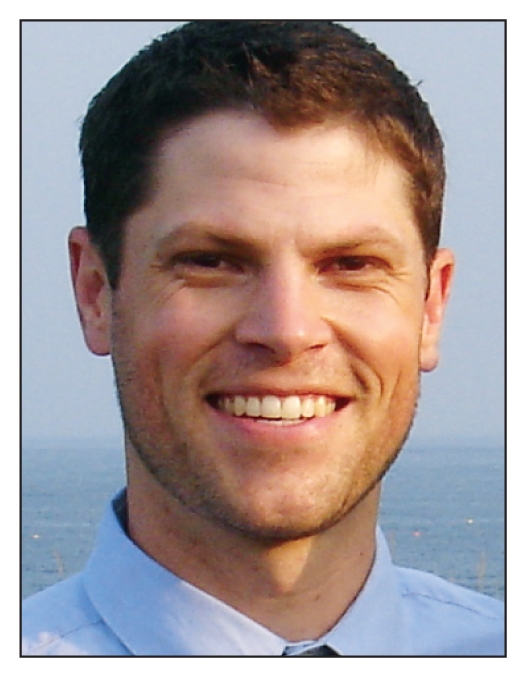
John Meeker

**Figure:**
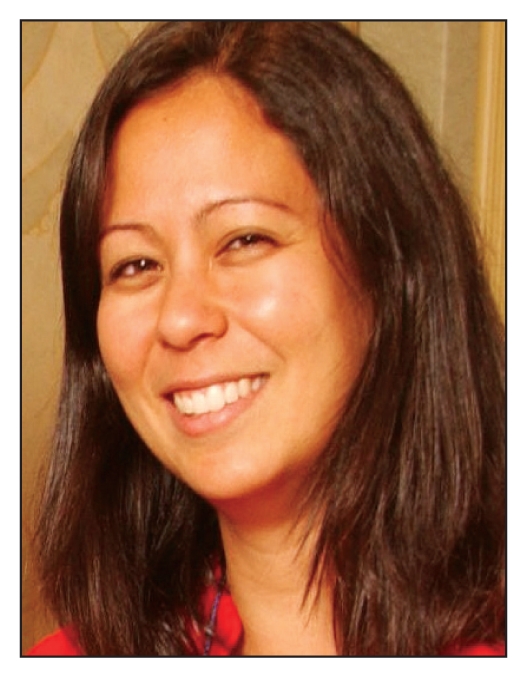
Sumi Mehta

**Figure:**
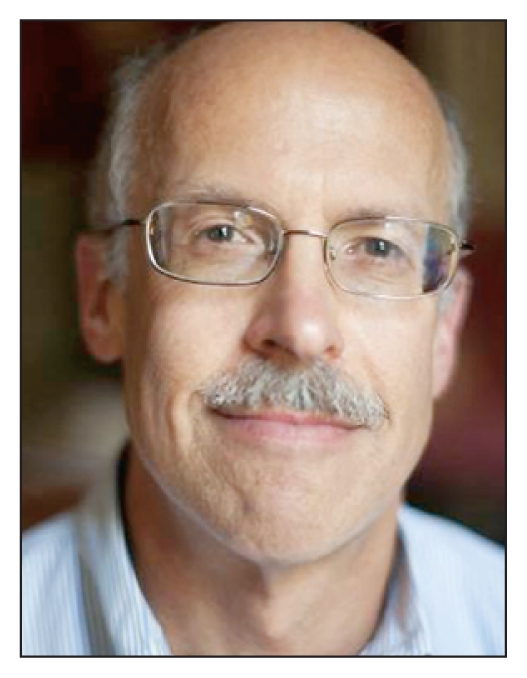
David Savitz

**Figure:**
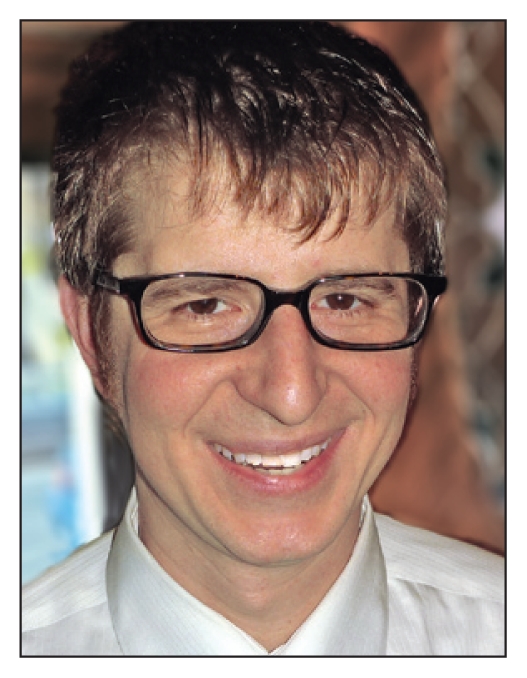
Leonardo Trasande

**Figure:**
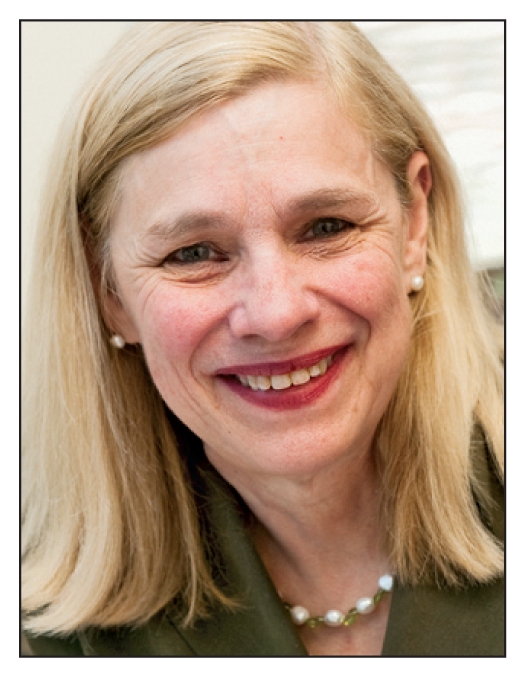
Roberta White

**Figure:**
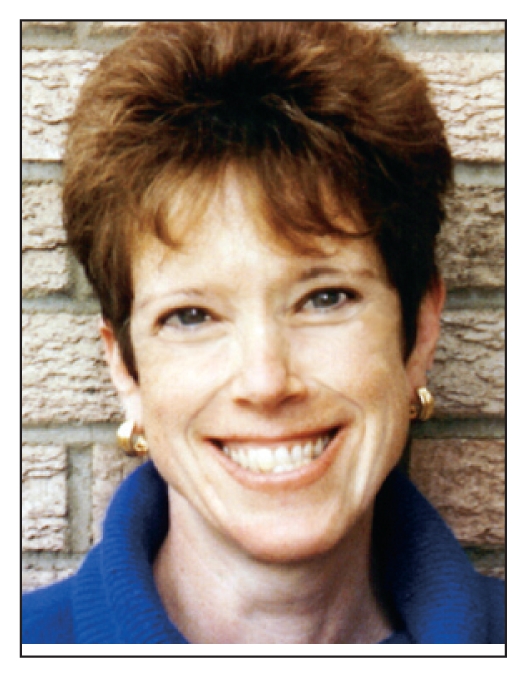
Judith Zelikoff

